# Development of an international external quality assurance program for HIV-1 incidence using the Limiting Antigen Avidity assay

**DOI:** 10.1371/journal.pone.0222290

**Published:** 2019-09-16

**Authors:** Sheila M. Keating, Wes Rountree, Eduard Grebe, Andrea L. Pappas, Mars Stone, Dylan Hampton, Christopher A. Todd, Marek S. Poniewierski, Ana Sanchez, Cassandra G. Porth, Thomas N. Denny, Michael P. Busch, for the EQAPOL Limiting Antigen (LAg) Incidence Assay External Quality Assurance (EQA) Program

**Affiliations:** 1 Vitalant Research Institute, San Francisco, CA, United States of America; 2 Department of Laboratory Medicine, University of California, San Francisco, CA, United States of America; 3 Duke Human Vaccine Institute, Duke University, Durham, NC, United States of America; Food and Drug Administration, UNITED STATES

## Abstract

Laboratory assays for identifying recent HIV-1 infections are widely used for estimating incidence in cross-sectional population-level surveys in global HIV-1surveillance. Adequate assay and laboratory performance are required to ensure accurate incidence estimates. The NIAID-supported External Quality Assurance Program Oversight Laboratory (EQAPOL) established a proficiency testing program for the most widely-used incidence assay, the HIV-1 Limiting Antigen Avidity EIA (LAg), with US Centers for Disease Control and Prevention (CDC)-approved kits manufactured by Sedia Biosciences Corporation and Maxim Biomedical. The objective of this program is to monitor the performance of participating laboratories. Four rounds of blinded external proficiency (EP) panels were distributed to up to twenty testing sites (7 North American, 5 African, 4 Asian, 2 South American and 2 European). These panels consisted of ten plasma samples: three blinded well-characterized HIV-1-seropositive samples that were included as replicates and an HIV-negative control. The seropositive samples spanned the dynamic range of the assay and are categorized as either recent or long-term infection. Participating sites performed the assay according to manufacturers’ instructions and completed an online survey to gather information on kit manufacturer, lot of kit used, laboratory procedures and the experience of technicians. On average, fifteen sites participated in each round of testing, with an average of four sites testing with only the Maxim assay, seven testing with only the Sedia assay and five sites utilizing both assays. Overall, the Sedia and Maxim assays yielded similar infection status categorization across the laboratories; however, for most of the nine HIV+ samples tested, there were significant differences in the optical density readouts, ODn (N = 8) and OD (N = 7), between LAg kit manufacturers (p < 0.05 based on mixed effects models. The EQAPOL LAg program is important for monitoring laboratory performance as well as detecting variations between manufacturers of HIV-1incidence assays.

## Introduction

Assays to identify recent HIV infection have revolutionized the science of incidence calculation and surveillance[1–3]. HIV prevention programs rely on accurate and precise estimates of incidence to measure an intervention program’s impact on HIV transmission and to guide resource allocation to optimize and improve HIV prevention [4]. While HIV incidence can be calculated from longitudinal HIV surveillance studies, they are expensive to support and prone to bias [2,5,6]. Cross-sectional incidence testing enables identification of recent infections in individuals who receive an HIV positive test result and is a more efficient way to calculate incidence in the surveillance population. In 1999, the STARHS algorithm was established, using a sensitive HIV antibody test to identify infection, followed by a less sensitive (LS) HIV antibody test to identify early HIV infection due to increasing antibody concentration during seroconversion [1,7–9]. Several additional incidence tests were developed and used in the US and at US-funded international sites, but it quickly became evident that assays were overestimating incidence calculations due to assay bias [10] in field studies [9], for reasons such as cohorts with treatment or natural control of HIV infection. New assays were introduced, using calibrators to control variability and avidity-modifications to minimize the impact of changes in antibody maturation due to treatment, natural control of viral replication (elite controllers) or low CD4 counts [11,12]. To monitor variability across sites, a CDC-directed proficiency program was initially rolled out to monitor assay performance and variability of the laboratory processes, but this program was later discontinued [13]. Harmonization of techniques to measure recent HIV infection, and to calculate incidence in populations, is essential for producing reliable and consistent results[3].

During the 2000s, the poor performance of the then front-line incidence assays BED and Vironostika led to the development of new assays such as the AxSYM and ACRHITECT Avidity, LS-VITROS and the HIV-1 Limiting Antigen Avidity EIA (LAg) for monitoring HIV incidence [14–18]. It was noted that a panel of reference samples was not available to assess within-assay and across-assay performance and to validate new assays prior to global distribution. In fact, there were no metrics developed to assess the performance or precision of the assay compared to known standards. In response to this, the Consortium for the Evaluation and Performance of HIV Incidence Assays (CEPHIA) was established and global specimens were curated and centrally located by the CEPHIA study group at Vitalant Research Institute (VRI, formerly Blood Systems Research Institute) to create panels of specimens to validate newly developed assays [19]. In addition, these same specimens could be used to validate new biomarkers or new technologies for more accurate incidence estimation [20]. To further refine the quality of results, systematic assessments of assay performance across US-funded testing labs was required to determine and enhance the consistency and accuracy of the surveillance results. In order to do this, the LAg Incidence Assay External Quality Assurance (EQA) program was established in 2015 within the EQAPOL program.

The goals of the EQAPOL LAg Incidence Assay EQA program are to assess the proficiency of NIAID/DAIDS- and CDC supported laboratories at performing an HIV-1 LAg incidence assay used in HIV incidence surveillance, to measure site performance consistency over time, and to monitor assay performance between manufacturers in order to identify variables that significantly impact assay outcomes. A scientific steering committee (SC) consisting of leaders in the field of HIV incidence was established for program guidance and oversight. One pilot round and four send-outs have been performed to date: January 2016 (EP1), January 2017 (EP2), January 2018 (EP3) and August 2018 (EP4). The primary objectives of EP1 and EP2 were to optimize logistics and assess the feasibility of a LAg EQA program and to develop grading criteria to evaluate the performance of participating sites. After EP2, the grading criteria were established and a detailed document outlining the criteria was distributed to the sites for reference prior to the EP3 send-out. Once EP3 was completed, the EQAPOL team, along with the LAg program’s steering committee, evaluated the effectiveness of the criteria at measuring site performance and identifying sites requiring feedback or remediation discussions. We found areas that were not identified by the EP3 grading scheme and therefore updated the criteria prior to EP4.

EQAPOL provided sites with blinded panels of plasma specimens from HIV-positive and HIV-negative subjects to be assayed using the commercially available Sedia and/or Maxim LAg kits. Depending on site requirements in the future, the program could be extended to other HIV incidence surveillance or individual patient infection staging methods. The Sedia and Maxim LAg assays generated an Optical Density (OD) reading that was normalized to ODn by dividing the OD values by the reactivity of a kit-provided calibrator sample. As part of the testing algorithm, ODn values that fell below 2.0 required additional replicate confirmatory testing with the LAg assay. For ODn values less than 0.4, additional diagnostic serology testing was required to confirm anti-HIV positivity using the site-specific serology testing assay. The sites were not required to perform viral load testing. The EQAPOL LAg EQA specimen panel was sent to testing laboratories and measurements were collected through the EQAPOL web portal with assay-specific metadata captured through online surveys. These data were analyzed across four evaluations to determine the laboratory assay performance in sample classification, assay precision and other metrics such as protocol adherence or result reporting timeliness.

The data presented in this article are available publicly from the Duke Digital Repository (doi:10.7924/r4ff3r13q).

## Materials and methods

### The LAg EQA program

The EQAPOL team worked closely with the CDC to re-establish a quality assurance program that had previously been employed by CDC-funded HIV surveillance sites. This new program, under EQAPOL, would facilitate unbiased (i.e., independent of CDC which developed and promulgated the BED and LAg assays) monitoring for the sites performing HIV incidence surveillance. An interactive web-based system was created for integrating data from the testing sites to allow cross-site analysis by the central laboratory and EQAPOL statistical team. Each EP concept is developed into a comprehensive study plan, which is reviewed and approved by EQAPOL leadership and the EQAPOL quality assurance team. The Study Plan contains an overview of EP organization and management, list of participating sites, proficiency panel design, assay details, data submission process, confidentiality protections, statistical analysis plan, and results reporting process. Attachments to the study plan include a list of selected samples from the repository, a confidential data key for the web-application to link coded assay results to sample identifiers in the database, a detailed kit-specific assay protocol, data submission templates, and EP orientation and training materials. Samples are pre-tested in the EQAPOL LAg Oversite Laboratory (EOL) using kits from both manufacturers to confirm protocol validity and sample homogeneity.

Laboratories that routinely perform the LAg incidence assay and that participated in the original CDC EQA program received an invitation to participate in the EQAPOL LAg Program including, by the time of the fourth evaluation panel (EP4), twenty active sites consisting of 7 North American, 5 African, 4 Asian, 2 South American and 2 European laboratories. Sites involved in the EQAPOL LAg program included the EOL at Duke, VRI, and the other sites listed in S1 Table. Sites using the Sedia and/or Maxim kits were invited to participate and were instructed to run the samples according to their kit-specific protocols and testing algorithm for initial/confirmatory testing. In addition, sites performed serology testing (but not viral load testing) when applicable using their site-specific testing procedures for HIV serology. Other assays or kit types have not yet been incorporated into this QA program, but the program may be expanded to include new incidence assays in future EPs.

### The LAg assay

Sites were required to perform the LAg assays according to manufacturer's instructions. In brief, assay controls and HIV-positive specimens were diluted 1:100 in specimen diluent and 100 μL of calibrator, controls or specimens were added to appropriate wells of antigen-coated plates and incubated for 60 min at 37°C. Plates were washed 4 times with 1x wash buffer to remove unbound antibodies. A pH 3.0 buffer was added to each well and incubated for 15 min at 37°C to dissociate low avidity antibodies. Washed plates were incubated with anti-human IgG peroxidase (30 min at 37°C), then washed and incubated with tetramethyl benzidine substrate (15 min at 25°C). Color development was stopped by addition of 100 μL/well of 1N H2SO4. The optical density (OD) was read at 450 nm with 650 nm as a reference using a spectrophotometer. Raw OD for each specimen was normalized using the calibrator (CAL) OD on each plate as a ratio, such that normalized OD (ODn) = (OD of specimen/median OD of CAL). Negative and positive controls are tested on every plate and determined to be within range to confirm the validity of the plate.

### The LAg EQA evaluation panels

Through routine blood screening, blood collections that were identified as HIV viral load and antibody positive units were quarantined from transfusion and used as part of this and other research studies. The large volume plasma units were tested across available incidence assays and found to span the dynamic range of the assays and were categorized as derived from donors with recent to long-term HIV infections. The units were sub-aliquoted, blinded, and incorporated into external proficiency panels (EPs). In EP1 and EP2, the sample kits include three blinded HIV-positive samples (each represented in separately coded triplicates) for a total of nine samples plus one HIV-negative control sample. The samples included in the EPs were well-characterized in terms of the repeatability of results from multiple testing rounds using both manufacturers LAg assays and many other incidence assays and determined to be consensus recent, long-term or negative based testing from multiple laboratories. The categorizations are as follows: 0.1–0.5 ODn (recent low), 0.5–1.5 ODn (recent high), and 1.5 ODn or higher is long-term. The ranges selected were based on a recommendation from the EQAPOL Scientific Advisory Board (SAB) to include samples that would not be too close to the 1.5 ODn cutoff value for recent and long-term. New samples were selected for EP3 by screening eighty additional HIV+ donors for potential sample candidates. After down selection, twelve candidate samples were tested eight times on different assay runs and on two different kit platforms to generate sixteen data points for each sample. These data were used to confirm the characterization of the sample in terms of status of infection (recent, long-term or negative) and the expected ODn value, as well as to measure the variability of the sample ODn values. Three samples that displayed the least amount of variability, and that did not change infection status in any of the sixteen assay runs from each category, were selected. In EP4, sites received three samples that were near the 1.5 ODn cutoff value for recent and long-term. These samples were not used for grading but were used to further investigate potential differences between the Sedia and Maxim kits. All samples were collected according to protocols approved by the Duke University Medical Center Institutional Review Board and UCSF Committee for Human Research.

#### Shipping of materials to participating laboratories

For each EP, the EQAPOL and VRI teams provided the sites with a proficiency testing kit consisting of: 1) 100μL aliquots of blinded samples to be stored at -80°C upon receipt; 2) assay instructions, assay questionnaire, and assay data reporting sheet (XLS) provided on the EQAPOL web-based system; 3) a data logger (Delta-TRAK) to monitor the temperature of the shipment while in transit and instructions on how to send it back; 4) timeline for completion of the testing and submission of data via the web-based application. No other reagents were provided to the sites in the send-out; sites procured and used their own assay kits/reagents for the LAg and serology assays. Sites were given the option to receive one or two sets of samples based on whether they normally perform testing with only Sedia or Maxim LAg or both and volume was sufficient for confirmatory serological testing.

#### Data reporting and grading criteria

Data was reported to the EQAPOL EOL via the web-based application using the Excel template provided by the EQAPOL team. The requested information includes calibrator OD values, sample ODn values, sample classifications, and serology status where required by the algorithm. The kit type (Sedia and/or Maxim) was also recorded.

Sites were graded according to five sets of criteria as outlined in Table 1 and described briefly below.

**Table 1:** Proficiency criteria and average scores.

Grading Criteria and Points Allocation			Average Scores					
			EP2 *retrospectively graded*		EP3		EP4	
**Criterion**	Points in EP3	Points in EP4	Sedia (N = 11)	Maxim (N = 8)	Sedia (N = 13)	Maxim (N = 8)	Sedia (N = 12)	Maxim (N = 9)
**Timeliness**	**10** *10 points received for on-time submission*	**10** *10 points received for on-time submission*	9.1	7.5	10	10	10	10
**Protocol Adherence**	**10** *4 points lost per protocol deviation*	**24** *4 points lost per protocol deviation*	8.2	10	10	10	24	23.6
**Classification**	**60** *15 points lost per incorrect call*	**48** *12 points lost per incorrect call*	50.5	54.4	60	60	45	46.7
**Precision**	**20** *3 points lost per* *sample type deviation*	**18** *3 points lost per* *sample type* *deviation*	15.1	13.3	14.9	14	12.3	12.7
**Accuracy to Consensus**								
**Total:**			**82.8**	**85.1**	**95**	**94**	**91.3**	**92.9**

#### Timeliness

Sites were given four weeks from kit receipt to complete the assay and return the data reporting excel files and post-assay questionnaire. Failure to upload valid data and survey responses by the due date resulted in a site’s loss of all proficiency points for timeliness (10 points).

#### Protocol adherence

Sites were assessed for their ability to follow the protocol and Excel Macro provided by EQAPOL. Sites received a four-point deduction for each deviation from the protocol and Excel Macro algorithm. During EP3 this category was worth ten points and was updated for EP4 to be worth twenty-four points. Deviations include but are not limited to: neglecting to perform serology or confirmatory testing when prompted by the protocol and Excel algorithm and incorrectly performing one of the four incubations defined by the protocol.

#### Recency status classifications

The primary objective of the LAg program send outs were to assess a site’s ability to properly classify a sample as HIV-negative, recent infection, or long-term infection. During EP3, each misclassification resulted in a fifteen-point deduction per sample with a total of sixty points allotted to this category. The point allotment was updated prior to EP4 for each misclassification resulting in a twelve-point deduction with a total of forty-eight points in this category.

#### Precision of results

Precision describes the amount of variability between repeated measures under unchanged conditions (i.e., the same operator, same instrument). As the field has begun to use the LAg Incidence Assay in a more quantitative manner, it has become important to assess the reliability of ODn values reported by sites. Precision was assessed by looking at the variability of ODn values between replicate samples for a given sample. The site variance was compared to the overall variance of all sites for that kit type and sample. Results that were significantly different from the model-based variance estimates incurred a three-point deduction with a maximum reduction of eighteen points. During EP3 this category was worth twenty points.

#### Accuracy of quantitative results (compared to consensus)

Accuracy is defined as the closeness of an estimate to either a true (known) value or an accepted reference standard. While there is a true classification for a sample for the Incidence Assay, there is not a true ODn value, so EQAPOL uses the consensus average as the accepted reference. Accuracy was assessed for each sample based on a comparison of the site’s average of the replicate sample’s ODn values to the consensus average for all sites reporting data for that kit type and sample. Results that were significantly different from the model-based consensus average incurred a three-point deduction with a maximum reduction of eighteen points. During EP3 this category was worth twenty points.

### Statistical analyses

The data collected in the EQAPOL LAg Program are analyzed according to statistical approaches currently in use for other EQAPOL programs [21]. The primary statistical approach is the use of mixed effects models and specific analyses are described. The comparisons, made for kit type means and variances, were performed in SAS 9.4 using Proc Mixed to calculate estimates from mixed effects models. The models for the mean comparisons had the OD or ODn values as the outcome variable with fixed effects for kit type and an interaction of kit type with the sample ID. These models also had a random intercept for each site by sample ID interaction with a grouping on EP. The models for the variance comparisons had the OD or ODn values as the outcome variable with a fixed effect for EP. These models also had a random intercept for each site while grouping on kit type by sample ID interaction. The model for the calibrator mean comparison had the calibrator OD value as the outcome variable with fixed effects for EP, kit type, and testing ‘mode’ with a random intercept for each site. The model for the calibrator variance comparison had the calibrator OD value as the outcome variable with fixed effects for EP and testing mode with a random intercept for each site grouping on kit type. For these descriptive comparisons we set the alpha level at 0.05 with no adjustments for multiple comparisons.

## Results

### Site performance

For each EP, summary results were calculated from the reported classifications (recent or long-term HIV+ infection or negative), calibrator OD and test sample OD/ODn. EP1 and EP2 results were initially graded using pass/fail criteria. The grading criteria for evaluating participating sites were developed based on observed performance in EP1 and EP2 and then implemented for EP3 and EP4, with EP2 also being retrospectively graded (see Table 1). There was an improvement in the overall average points between EP2 and EP3. After EP3, the grading criteria were modified to better identify sites with assay performance issues and sites that could benefit from remediation efforts. We discovered site performance issues that were not identified based on the EP3 grading criteria. Therefore, we modified the grading criteria in order to better capture some of the issues observed in EP3. The EP3 criteria and the changes implemented in EP4 as well as average results for each criterion and overall average scores are outlined in Table 1. Since the grading criteria were adjusted in EP4, there is no clear way to directly compare the scoring over time.

### Stability of results over time

Recall that in EPs 1 and 2 the same three samples (LA_0001, LA_0006 and LA_0009) were sent to the sites for testing. Similarly, in EPs 3 and 4 a new set of three samples were sent to the sites for testing including samples LA_0002, LA_0003 and LA_0004. In EP4 three additional samples near the standard ODn cutoof value of 1.5 were included in the panel (LA_0005, LA_0007 and LA_0008). These samples were not utilized for grading but were included in order to have a sample set spanning the range of OD and ODn values for more refined data analysis with particular interest in between-manufacturer performance comparisons, after differences were noticed in EPs 1–3. We evaluated the OD and ODn values obtained on the same samples over the EPs to see if there were shifts in reactivity over time. On the whole, results were very stable, with only one of the six samples (LA_0003), not near the cutoff showing a significant difference between EPs 3 and 4, based on mixed effects models for OD and ODn (see S2 and S3 Tables).

### Performance across kit manufacturers

**Fig 1:**
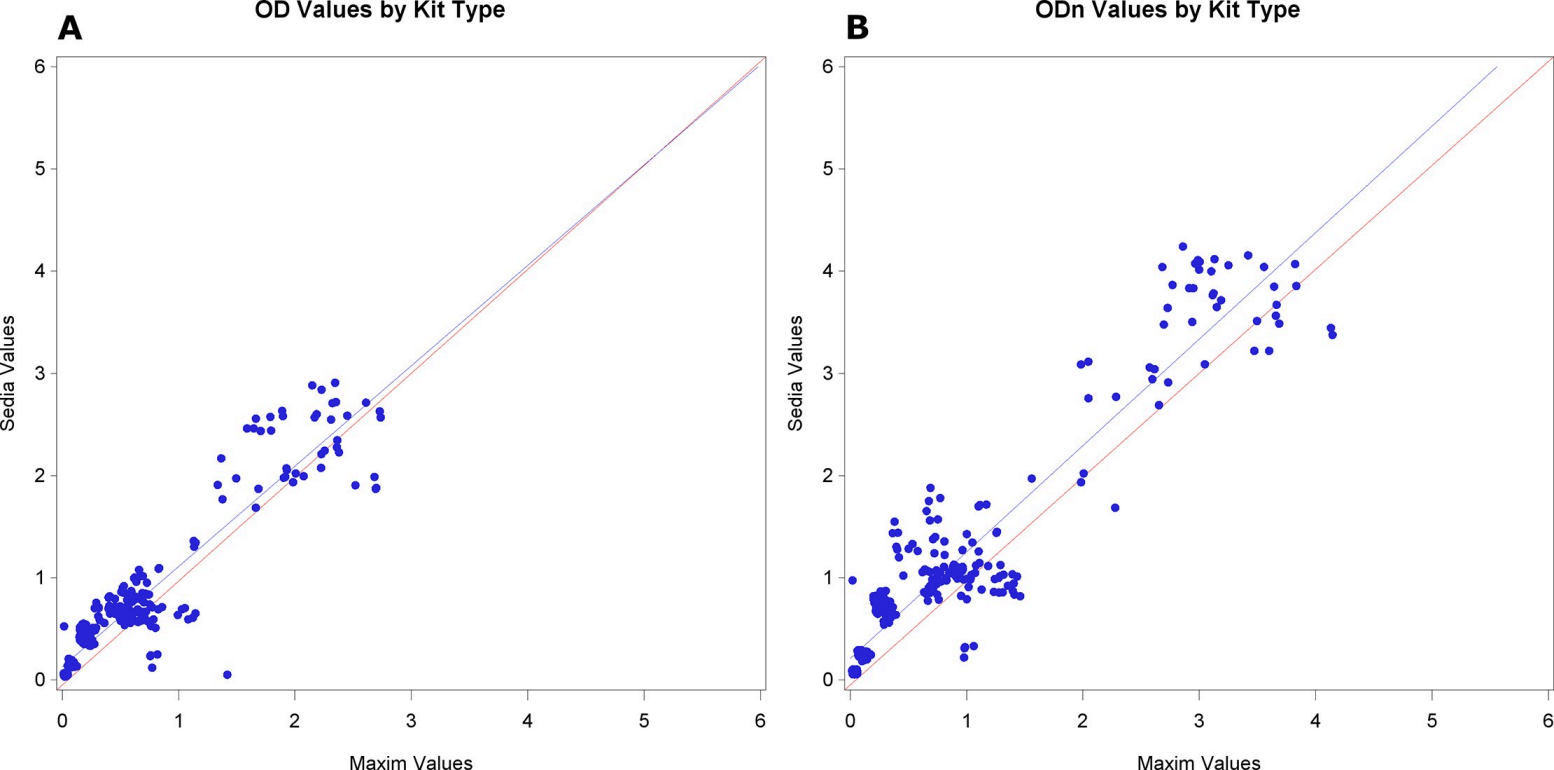
Comparison of Maxim and Sedia OD and ODn values. Panel A shows OD readings and Panel B shows ODn readings. The blue line is the linear regression line and the red line shows the slope if the two kits had produced equivalent results.

It is important to compare sample OD and ODn values obtained using kits from the two manufacturers since field laboratories could change manufactures over time. For both the Maxim and Sedia kits the recency classification of a sample is obtained by comparing the ODn value to a common ODn cutoff value of 1.5. The sample OD values are divided by the calibrator OD value to calculate the normalized ODn values. Calibrator specimens are supplied by the manufacturers with the kits. Fig 1A shows the raw OD values for the two kit types on the same specimens, and Fig 1B the normalized ODn values. A linear regression line in blue for the sample values obtained, and a slope of 1 (red line) that would be obtained if the two kits had produced identical results. These graphs indicate that the OD values are more similar than the ODn values when comparing results from Sedia and Maxim kits (regression slope is closer to unity). This result led us to consider whether the calibrator values for the two kits were different, since the ODn value is calculated using the calibrator. The difference in calibrator values for EPs 1–4 across all kits was compared using a mixed effects model and the mean Maxim value was 0.7225 and the mean Sedia value was 0.6859, with a 95% confidence interval of the difference (0.0096, 0.0637), which is significant (p = 0.0080). A separate mixed effects model was used to estimate the within and between site variance for OD and ODn values for each sample tested (S4 and S5 Tables). The within and between site variance estimates for the two kit calibrator values were also compared (Table 2), and there is evidence that the Maxim within-site kit calibrator variance is higher based on non-overlapping 95% confidence intervals. Given the fact that the overall mean Maxim calibrator value is higher than the Sedia calibrator value, a higher within site variance estimate is expected.

**Table 2:** Between-site and within-site variances for kit calibrators, EPs 1–4 (mixed effects model estimates).

	Model-Based Variance Mean (95% CI)		
Variance Type	Maxim Kit	Sedia Kit	Result
Between Site	0.00473 (0.00205, 0.02014)	0.00247 (0.00118, 0.00806)	Variances Equal
**Within Site**	**0.01425 (0.01166, 0.01781)**	**0.00940 (0.00791, 0.01135)**	**Maxim Var Higher**

Given the theoretical role of the calibrators is to control for differences in assay performance across sites and technicians and as kit lots age, there was further interest in evaluating the effect of the calibrators on the within and between site values. A mixed effects model was run using all data from EPs 1–4 to estimate the between and within variance estimates for Maxim and Sedia kits for OD and ODn values. This model adjusted for site, sample, and kit type and the intraclass correlation (ICC) was calculated. ODn variances are higher than the OD variances (Table 3), but given that the ODn values are higher on average, this is expected. However, to compare overall variance and evaluate whether the use of calibrators reduces variability, the ICC was calculated. The ICC is the ratio of the between-site variability to the total variability [ICC = σb / σb + σw (σb = between site variance; σw = within site variance]. The results of this model provided nearly identical ICC estimates for both OD and ODn values, on both kit types: Sedia OD ICC = 0.962 and the ODn ICC = 0.959, Maxim OD ICC = 0.949 and the ODn ICC = 0.967. We therefore found no evidence of a reduction in the between-site variance through the use of calibrators to normalize results (see Table 3).

**Table 3:** Comparison of between-site and within-site variances of OD and ODn values for EPs 1–4 (mixed effects models estimates).

		Model-Based Variance Mean (95% CI)			Intraclass Correlation	
Kit Type	Variance Type	OD Values	ODn Value	Result	OD ICC	ODn ICC
**Maxim**	**Between Site**	**0.4610 (0.3452, 0.6470)**	**0.9471 (0.7098, 1.3280)**	**ODn Var Higher**	.	.
**Maxim**	**Within Site**	**0.0246 (0.0221, 0.0274)**	**0.0321 (0.0290, 0.0358)**	**ODn Var Higher**	0.949	0.967
**Sedia**	**Between Site**	**0.6359 (0.4924, 0.8532)**	**1.5598 (1.2073, 2.0937)**	**ODn Var Higher**	.	.
**Sedia**	**Within Site**	**0.0249 (0.0227, 0.0275)**	**0.0666 (0.0608, 0.0734)**	**ODn Var Higher**	0.962	0.959
**Both**	**Between Site**	**0.5769 (0.4747, 0.7163)**	**1.3503 (1.1110, 1.6767)**	**ODn Var Higher**	.	.
**Both**	**Within Site**	**0.0248 (0.0231, 0.0266)**	**0.0514 (0.0479, 0.0552)**	**ODn Var Higher**	0.959	0.963

Variances adjusted for site, kit type, and sample.

**Fig 2:**
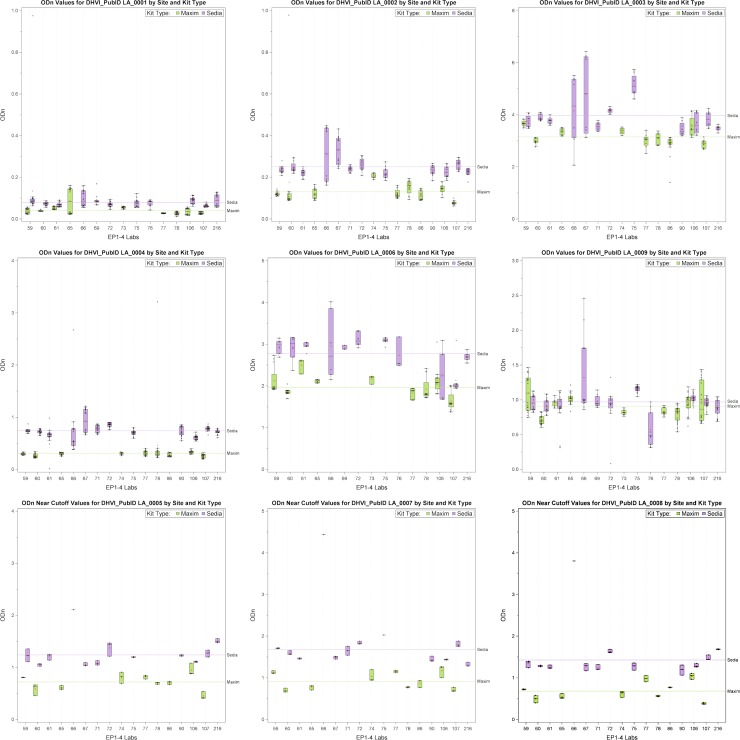
Mean ODn values for each sample by site and kit type. Maxim kits are shown in green and Sedia kits are in purple. Eight out of nine samples had significantly higher Sedia means than Maxim means.

Another important assessment of these LAg EP data is the comparison of the Maxim and Sedia average OD and ODn values. Mixed effects models were run using all data from EPs 1–4 to estimate the Maxim and Sedia mean OD values or ODn values to compare these values between kits for each sample. For OD values there were seven of the nine samples where the Sedia values were significantly higher than Maxim values (See Table 4). Similarly, for ODn values there were eight of the nine samples where the Sedia values were significantly higher than Maxim values (See Table 5). These mean shifts can be seen visually via the box and whisker plots for the ODn values (Fig 2) and OD values (S1 Fig). Note that the ratios of Sedia to Maxim results are similar for OD and ODn values. See Table 6 for a breakdown of the data provided by each lab.

**Table 4:** Comparison of average OD values by kit type for EPs 1–4 (mixed effects model estimates).

	Model-Based Mean (95% CI)				
ID	Sedia Mean	Maxim Mean	Ratio	Sedia—Maxim: Mean (95% CI)	p-value
LA_0001	0.0570	0.0247	2.3	0.0323 (-0.0003, 0.0649)	0.0518
**LA_0002**	**0.1744**	**0.1049**	**1.7**	**0.0694 (0.0258, 0.1131)**	**0.0019**
**LA_0003**	**2.5326**	**2.2283**	**1.1**	**0.3043 (0.2518, 0.3568)**	**< .0001**
**LA_0004**	**0.5061**	**0.2556**	**2.0**	**0.2505 (0.2143, 0.2867)**	**< .0001**
**LA_0005**	**0.7898**	**0.5420**	**1.5**	**0.2478 (0.1431, 0.3524)**	**< .0001**
**LA_0006**	**1.9825**	**1.6519**	**1.2**	**0.3306 (0.2836, 0.3776)**	**< .0001**
**LA_0007**	**1.0806**	**0.7015**	**1.5**	**0.3792 (0.2744, 0.4839)**	**< .0001**
**LA_0008**	**0.9355**	**0.5408**	**1.7**	**0.3947 (0.2900, 0.4993)**	**< .0001**
LA_0009	0.6608	0.6470	1.0	0.0138 (-0.0188, 0.0464)	0.4067

**Table 5:** Comparison of average ODn values by kit type for EPs 1–4 (mixed effects model estimates).

	Model-Based Mean (95% CI)				
ID	Sedia Mean	Maxim Mean	Ratio	Sedia—Maxim: Mean (95% CI)	p-value
**LA_0001**	**0.0847**	**0.0356**	**2.4**	**0.0490 (0.0045, 0.0936)**	**0.0309**
**LA_0002**	**0.2561**	**0.1369**	**1.9**	**0.1192 (0.0536, 0.1848)**	**0.0004**
**LA_0003**	**3.8752**	**3.2450**	**1.2**	**0.6302 (0.5507, 0.7097)**	**< .0001**
**LA_0004**	**0.7476**	**0.3238**	**2.3**	**0.4238 (0.3699, 0.4777)**	**< .0001**
**LA_0005**	**1.2574**	**0.7784**	**1.6**	**0.4790 (0.3235, 0.6345)**	**< .0001**
**LA_0006**	**2.7661**	**2.1959**	**1.3**	**0.5702 (0.5045, 0.6359)**	**< .0001**
**LA_0007**	**1.7778**	**1.0426**	**1.7**	**0.7352 (0.5796, 0.8908)**	**< .0001**
**LA_0008**	**1.5042**	**0.7928**	**1.9**	**0.7114 (0.5558, 0.8669)**	**< .0001**
LA_0009	0.9561	0.9630	1.0	-0.0069 (-0.0515, 0.0376)	0.7604

**Table 6:** Data summary of laboratories by kit type for EPs 1–4.

Site	Maxim EPs	Sedia EPs	Maxim Calibrators	Sedia Calibrators	Total Calibrators	Maxim OD Values	Sedia OD Values	Total OD
059	4	4	24	24	48	95	89	184
060	4	4	24	24	48	99	89	188
061	1	4	6	24	30	21	89	110
065	4		24		24	89		89
066		3		18	18		77	77
067		2		12	12		47	47
069		1		6	6		21	21
071		2		12	12		47	47
072		4		24	24		83	83
073	1		6		6	21		21
074	1		6		6	28		28
075		4		24	24		87	87
076		1		6	6		21	21
077	3		18		18	74		74
078	4		24		24	95		95
086	2		12		12	53		53
090		2		12	12		47	47
106	4	4	24	24	48	95	89	184
107	4	4	24	24	48	95	91	186
216		4		24	24		89	89
**Total**	**32**	**43**	**192**	**258**	**450**	**765**	**966**	**1731**

## Discussion

The EQAPOL LAg EQA program, which is jointly managed by Duke University and Vitalant Research Institute and is supported by the NIH/NIAID, has successfully launched an international EQA/proficiency testing program for the HIV-1 LAg assay. The assays utilized in this program are CDC-approved and widely employed globally to classify HIV infections as recently acquired or longstanding for the purposes of cross-sectionally estimating incidence in population-level studies [22–25]. Increasingly, expanded use-cases for individual-level infection staging and improved estimation of individual time of infection (in combination of diagnostic test results) are being contemplated and piloted. These new, currently unregulated, uses have potential clinical applications–individuals treated early in infection have improved clinical outcomes [26] and establish smaller latent HIV reservoirs [27]–and can inform public health interventions such as enhanced contact tracing [28]. Both the incidence surveillance and individual-level applications require highly consistent results between laboratories, manufacturers and over time.

The program is a result of collaborative efforts between EQAPOL, NIAID and the CDC and it currently monitors twenty domestic and international sites with two scored EP panels per year. The goal for the EQA testing program is to promote consistent and appropriate assay procedures and improve assay proficiency within participating laboratories. The comprehensive scoring allows for identification of sites who could benefit from additional training and remediation. Overall, the program promotes improvement through a combination of proficiency testing, questionnaires, remediation and training.

An important program goal is to develop and use proficiency testing to identify factors affecting LAg assay outcomes globally. Analysis of the four proficiency testing rounds showed differences between optical densities (OD) and normalized optical densities (ODn) from LAg kits from the two manufacturers, with Maxim-produced kits producing lower OD and ODn measurements than Sedia-manufactured kits, on average. Differences in ODn values were larger than those in OD values in absolute terms, but not in relative terms, since ODn are inflated relative to OD values by the normalization procedure and ratios of Sedia to Maxim OD readings and ODn readings were similar. However, the calibrators did appear to exacerbate differences to some extent, as evidenced by the lower correlation between ODn values than OD values produced using the two kit types, as shown in the regression analysis.

The purpose of the calibrators is to control for variability in laboratory procedures (such as incubation times and temperatures, input volumes, etc.) between sites, lot-to-lot variations and potential changes over time as kits age. We demonstrate significant differences in ODn results between the two kit types, a finding that is consistent with previous studies comparing the performance of the two manufacturers’ kits on the same specimens in a single laboratory, including the CEPHIA analysis of comparative performance reported in this journal [29,30] We further demonstrated significant differences in raw ODs measured by the two kits, which are not resolved by normalization with the kit-supplied calibrators. Pervious studies were not able to assess the impact of normalization on lot-to-lot and site-to-site variability. Our analysis of the impact of calibrators on between-site variance of ODn values failed to demonstrate an impact (positive or negative) compared to raw OD measurements. Further work is needed to investigate whether calibration successfully controls for kit and reagent degradation over time.

While these results do not show that the normalization procedure is harming performance, given the evidence that there are differences in the calibrators supplied by the two kit manufacturers, the benefit of the normalization procedure is called into question. While current instructions for use remain in place, users should be aware of the differences in OD results and in the calibrators when selecting kits, choosing a recency discrimination threshold, and when planning or analyzing survey data and reporting results. Users should avoid switching kit manufacturers during studies. To our knowledge, the LAg EQAPOL program provided the first evidence that the performance of the Sedia and Maxim assays differed. We demonstrated and communicated our findings after the pilot studies and early EPs, which led to studies by CEPHIA, CDC and John Hopkins University that confirmed the differences. It should be noted that changing assay procedures to remove calibrators would at best only partially address the differences, and would require recalibration of both assays (i.e., deriving new MDRI and FRR estimates). We therefore do not recommend a change in procedures at this stage.

The primary objective of the EQAPOL LAg EQA program is to support laboratories that provide patient screening and identify recent HIV infections. As we continue to develop the program and look to future needs in this field, we will work to incorporate EQA support for other incidence assays/testing platforms and sample types (e.g., Dried Blood Spots). As an example, we plan to integrate rapid point-of-care recency assays into the program as these assays provide a valuable option for obtaining faster results, which may support clinical and public health decision-making. We aim to help facilitate improvements in HIV screening, including ascertainment of recency, which will support advancements in universal testing, treatment, viral suppression of HIV and incidence reduction worldwide.

## Supporting information

S1 Table Active sites in the EQAPOL LAg EQA program. (DOCX)

S2 Table Comparison of mean OD shifts in EPs 1–4 (mixed effects model estimates). (DOCX)

S3 Table Comparisons of mean ODn shifts in EPs 1–4 (mixed effects model estimates). (DOCX)

S4 Table Comparison between Sedia and Maxim between-site and within-site variances of OD measurements for EPs 1–4 (mixed effects model estimates). (DOCX)

S5 Table Comparison between Sedia and Maxim between-site and within-site variances of ODn measurements for EPs 1–4 (mixed effects model estimates). (DOCX)

S1 Fig Mean OD values for each sample by site and kit type. Maxim kits are shown in green and Sedia kits are in purple. Eight out of nine samples had significantly higher Sedia means than Maxim means.
(TIF)

## References

[1] Janssen RS, Satten GA, Stramer SL, Rawal BD, O’Brien TR, Weiblen BJ, et al New Testing Strategy to Detect Early HIV-1 Infection for Use in Incidence Estimates for Clinical and Prevention Purposes. JAMA. 1998;281: 1893. 10.1001/jama.280.1.42 9660362

[2] Busch MP, Pilcher CD, Mastro TD, Kaldor J, Vercauteren G, Rodriguez W, et al Beyond detuning: 10 years of progress and new challenges in the development and application of assays for HIV incidence estimation: AIDS. 2010;24: 2763–2771. 10.1097/QAD.0b013e32833f1142 20975514

[3] Murphy G, Pilcher CD, Keating SM, Kassanjee R, Facente SN, Welte A, et al Moving towards a reliable HIV incidence test–current status, resources available, future directions and challenges ahead. Epidemiology and Infection. 2017;145: 925–941. 10.1017/S0950268816002910 28004622 PMC9507805

[4] Brookmeyer R. Measuring the HIV/AIDS Epidemic: Approaches and Challenges. Epidemiologic Reviews. 2010;32: 26–37. 10.1093/epirev/mxq002 20203104

[5] Beyrer C, Nelson K. Loss to follow-up effect in investigations of HIV-1 incidence. 1997;349: 649–650. 10.1016/S0140-6736(05)61594-2 9057751

[6] Hallett TB, Ghys P, Bärnighausen T, Yan P, Garnett GP. Errors in ‘BED’-Derived Estimates of HIV Incidence Will Vary by Place, Time and Age. Galvani AP, editor. PLoS ONE. 2009;4: e5720 10.1371/journal.pone.0005720 19479050 PMC2684620

[7] Highleyman L. Detuned assay used to track recent infections. BETA. 1999;12: 6, 78. 11367259

[8] Rawal B, Degula A, Lebedeva L, Janssen R, Hecht F, Sheppard H, et al Development of a New Less-Sensitive Enzyme Immunoassay for Detection of Early HIV-1 Infection. Jaids Journal of Acquired Immune Deficiency Syndromes. 2003;33: 349–355. 12843746 10.1097/00126334-200307010-00009

[9] Parekh BS, Hanson DL, Hargrove J, Branson B, Green T, Dobbs T, et al Determination of Mean Recency Period for Estimation of HIV Type 1 Incidence with the BED-Capture EIA in Persons Infected with Diverse Subtypes. AIDS Research and Human Retroviruses. 2011;27: 265–273. 10.1089/aid.2010.0159 20954834

[10] Laeyendecker O, Rothman RE, Henson C, Horne BJ, Ketlogetswe KS, Kraus CK, et al The Effect of Viral Suppression on Cross-Sectional Incidence Testing in the Johns Hopkins Hospital Emergency Department: JAIDS Journal of Acquired Immune Deficiency Syndromes. 2008;48: 211–215. 10.1097/QAI.0b013e3181743980 18520680 PMC2738975

[11] Murphy G, Charlett A, Osner N, Gill ON, Parry JV. Reconciling HIV incidence results from two assays employed in the serological testing algorithm for recent HIV seroconversion (STARHS). Journal of Virological Methods. 2003;113: 79–86. 10.1016/s0166-0934(03)00222-2 14553893

[12] Young CL, Hu DJ, Byers R, Vanichseni S, Young NL, Nelson R, et al Evaluation of a Sensitive/Less Sensitive Testing Algorithm Using the bioMérieux Vironostika-LS Assay for Detecting Recent HIV-1 Subtype B’ or E Infection in Thailand. AIDS Research and Human Retroviruses. 2003;19: 481–486. 10.1089/088922203766774522 12882657

[13] Mei JV, Kennedy M, Linley L, Hanson D, Schiffer J, Ethridge S, et al Standardization and Monitoring of Laboratory Performance and Quality Assurance by Use of the Less-Sensitive HIV Incidence Assay: Seven Years of Results. J Acquir Immune Defic Syndr. 2011;58: 7. 10.1097/QAI.0b013e318230dd77 21857352

[14] Murphy G, Parry JV. Assays for the detection of recent infections with human immunodeficiency virus type 1. Euro Surveill. 2008;13. 18775293

[15] Suligoi B, Massi M, Galli C, Sciandra M, Di Sora F, Pezzotti P, et al Identifying recent HIV infections using the avidity index and an automated enzyme immunoassay. J Acquir Immune Defic Syndr. 2003;32: 424–428. 12640201 10.1097/00126334-200304010-00012

[16] Suligoi B, Rodella A, Raimondo M, Regine V, Terlenghi L, Manca N, et al Avidity Index for anti-HIV antibodies: comparison between third- and fourth-generation automated immunoassays. J Clin Microbiol. 2011;49: 2610–2613. 10.1128/JCM.02115-10 21543577 PMC3147844

[17] Keating SM, Hanson D, Lebedeva M, Laeyendecker O, Ali-Napo NL, Owen SM, et al Lower-Sensitivity and Avidity Modifications of the Vitros Anti-HIV 1+2 Assay for Detection of Recent HIV Infections and Incidence Estimation. Journal of Clinical Microbiology. 2012;50: 3968–3976. 10.1128/JCM.01454-12 23035182 PMC3503010

[18] Duong YT, Qiu M, De AK, Jackson K, Dobbs T, Kim AA, et al Detection of Recent HIV-1 Infection Using a New Limiting-Antigen Avidity Assay: Potential for HIV-1 Incidence Estimates and Avidity Maturation Studies. Landay A, editor. PLoS ONE. 2012;7: e33328 10.1371/journal.pone.0033328 22479384 PMC3314002

[19] Kassanjee R, Pilcher CD, Keating SM, Facente SN, McKinney E, Price MA, et al Independent assessment of candidate HIV incidence assays on specimens in the CEPHIA repository: AIDS. 2014;28: 2439–2449. 10.1097/QAD.0000000000000429 25144218 PMC4210690

[20] Kassanjee R, Welte A, McWalter TA, Keating SM, Vermeulen M, Stramer SL, et al Seroconverting Blood Donors as a Resource for Characterising and Optimising Recent Infection Testing Algorithms for Incidence Estimation. Gray CM, editor. PLoS ONE. 2011;6: e20027 10.1371/journal.pone.0020027 21694760 PMC3111407

[21] Rountree W, Vandergrift N, Bainbridge J, Sanchez AM, Denny TN. Statistical methods for the assessment of EQAPOL proficiency testing: ELISpot, Luminex, and Flow Cytometry. Journal of Immunological Methods. 2014;409: 72–81. 10.1016/j.jim.2014.01.007 24456626 PMC4104253

[22] Blaizot S, Kim AA, Zeh C, Riche B, Maman D, De Cock KM, et al Estimating HIV Incidence Using a Cross-Sectional Survey: Comparison of Three Approaches in a Hyperendemic Setting, Ndhiwa Subcounty, Kenya, 2012. AIDS Res Hum Retroviruses. 2017;33: 472–481. 10.1089/AID.2016.0123 27824254 PMC6779630

[23] Kim AA, Rehle T. Short Communication: Assessing Estimates of HIV Incidence with a Recent Infection Testing Algorithm That Includes Viral Load Testing and Exposure to Antiretroviral Therapy. AIDS Res Hum Retroviruses. 2018;34: 863–866. 10.1089/AID.2017.0316 29926735

[24] Soodla P, Simmons R, Huik K, Pauskar M, Jogeda E-L, Rajasaar H, et al HIV incidence in the Estonian population in 2013 determined using the HIV-1 limiting antigen avidity assay. HIV Med. 2018;19: 33–41. 10.1111/hiv.12535 28762652

[25] Justman JE, Mugurungi O, El-Sadr WM. HIV Population Surveys—Bringing Precision to the Global Response. New England Journal of Medicine. 2018;378: 1859–1861. 10.1056/NEJMp1801934 29768142

[26] Sharma S, Schlusser KE, Torre PDL, Tambussi G, Draenert R, Pinto AN, et al The benefit of immediate compared to deferred ART on CD4+ Cell count recovery in early HIV infection. AIDS. 2019; 10.1097/QAD.0000000000002219 31157663 PMC6561661

[27] Ostrowski M, Benko E, Yue FY, Kim CJ, Huibner S, Lee T, et al Intensifying Antiretroviral Therapy With Raltegravir and Maraviroc During Early Human Immunodeficiency Virus (HIV) Infection Does Not Accelerate HIV Reservoir Reduction. Open Forum Infect Dis. 2015;2: ofv138 10.1093/ofid/ofv138 26512359 PMC4621663

[28] Murphy G, Pilcher CD, Keating SM, Kassanjee R, Facente SN, Welte A, et al Moving towards a reliable HIV incidence test—current status, resources available, future directions and challenges ahead. Epidemiol Infect. 2017;145: 925–941. 10.1017/S0950268816002910 28004622 PMC9507805

[29] Schlusser KE, Konikoff J, Kirkpatrick AR, Morrison C, Chipato T, Chen P-L, et al Short Communication: Comparison of Maxim and Sedia Limiting Antigen Assay Performance for Measuring HIV Incidence. AIDS Research and Human Retroviruses. 2017;33: 555–557. 10.1089/aid.2016.0245 28318310 PMC5467096

[30] Sempa JB, Welte A, Busch MP, Hall J, Hampton D, et al Performance comparison of the Maxim and Sedia Limiting Antigen Avidity assays for HIV incidence surveillance. PLOS ONE 14(7): e0220345 10.1371/journal.pone.0220345 31348809 PMC6660077

