# Supplementary material for: Development of an international external quality assurance program for HIV-1 incidence using the Limiting Antigen Avidity assay
Source: PLoS One. 2019 Sep 16;14(9):e0222290. doi: 10.1371/journal.pone.0222290 (PMC6746377; doi:10.1371/journal.pone.0222290)
Supplement: S1 Table — (DOCX) [file pone.0222290.s001.docx]

S1 Table. Active sites in the EQAPOL LAg EQA program.

| **Site Name** | **City** | **Country** |
| --- | --- | --- |
| Centers for Disease Control and Prevention | Atlanta GA | USA |
| Johns Hopkins Medical Institute | Baltimore, MD | USA |
| Public Health England | London | United Kingdom |
| HIV Sero-Molecular Laboratory | Johannesburg | South Africa |
| Bangkok Metropolitan Administration | Nonthaburee | Thailand |
| Oswaldo Cruz Institute - FIOCRUZ | Rio de Janeiro | Brazil |
| AFRIMS | Rajchatheevee, Bangkok | Thailand |
| Botswana-Harvard AIDS Institute | Gaborrone | Botswana |
| Tropical Diseases Research Centre | Ndola | Zambia |
| National HIV Reference Laboratory | Nairobi | Kenya |
| KEMRI-CDC | Kisumu | Kenya |
| National Institute of Health | Nonthaburi | Thailand |
| Hospital Universitario Germans Trias | Badalona, Barcelona | Spain |
| Laboratório de Pesquisa em HIV/AIDS | Caxias do Sul, | Brazil |
| Maxim Biomedical | Rockville, MD | USA |
| Key Laboratory of AIDS Immunology | Shenyang City | China |
| Sedia Biosciences Corporation | Portland , OR | USA |
| EQAPOL Oversite Laboratory | Durham, NC | USA |
| Vitalant Research Institute | San Francisco, CA | USA |
| National HIV and Retroviology Laboratories | Winnipeg | Canada |
