# Supplementary material for: Development of an international external quality assurance program for HIV-1 incidence using the Limiting Antigen Avidity assay
Source: PLoS One. 2019 Sep 16;14(9):e0222290. doi: 10.1371/journal.pone.0222290 (PMC6746377; doi:10.1371/journal.pone.0222290)
Supplement: S2 Table — (DOCX) [file pone.0222290.s002.docx]

S2 Table. Comparison of mean OD shifts in EPs 1-4 (mixed effects model estimates).

|  | | **Model-Based Means (95% CI of the Difference)** | | |  |
| --- | --- | --- | --- | --- | --- |
| **ID** | **EPs** | **First EP** | **Second EP** | **First - Second: Mean (95% CI)** | **p-value** |
| LA_0001 | 1 vs 2 | 0.0383 | 0.0499 | -0.0117 (-0.1719, 0.1486) | 0.8860 |
| LA_0006 | 1 vs 2 | 1.8207 | 1.8064 | 0.0143 (-0.1496, 0.1783) | 0.8631 |
| LA_0009 | 1 vs 2 | 0.6334 | 0.7280 | -0.0946 (-0.2550, 0.0658) | 0.2459 |
| LA_0002 | 3 vs 4 | 0.1527 | 0.1212 | 0.0315 (-0.0760, 0.1390) | 0.5634 |
| **LA_0003** | **3 vs 4** | **2.4484** | **2.2772** | **0.1712 ( 0.0610, 0.2815)** | **0.0025** |
| LA_0004 | 3 vs 4 | 0.3960 | 0.3587 | 0.0374 (-0.0682, 0.1430) | 0.4857 |
