# Supplementary material for: Development of an international external quality assurance program for HIV-1 incidence using the Limiting Antigen Avidity assay
Source: PLoS One. 2019 Sep 16;14(9):e0222290. doi: 10.1371/journal.pone.0222290 (PMC6746377; doi:10.1371/journal.pone.0222290)
Supplement: S3 Table — (DOCX) [file pone.0222290.s003.docx]

S3 Table. Comparisons of mean ODn shifts in EPs 1-4 (mixed effects model estimates).

|  | | **Model-Based Means (95% CI of the Difference)** | | |  |
| --- | --- | --- | --- | --- | --- |
| **ID** | **EPs** | **First EP** | **Second EP** | **First - Second: Mean (95% CI)** | **p-value** |
| LA_0001 | 1 vs 2 | 0.0564 | 0.0695 | -0.0131 (-0.1919, 0.1657) | 0.8850 |
| LA_0006 | 1 vs 2 | 2.5008 | 2.4356 | 0.0652 (-0.1189, 0.2493) | 0.4855 |
| LA_0009 | 1 vs 2 | 0.9505 | 0.9826 | -0.0321 (-0.2113, 0.1471) | 0.7238 |
| LA_0002 | 3 vs 4 | 0.2045 | 0.1820 | 0.0225 (-0.2388, 0.2838) | 0.8653 |
| **LA_0003** | **3 vs 4** | **3.4514** | **3.7435** | **-0.2921 (-0.5557, -0.0286)** | **0.0300** |
| LA_0004 | 3 vs 4 | 0.5320 | 0.5425 | -0.0106 (-0.2704, 0.2492) | 0.9361 |
