# Supplementary material for: Development of an international external quality assurance program for HIV-1 incidence using the Limiting Antigen Avidity assay
Source: PLoS One. 2019 Sep 16;14(9):e0222290. doi: 10.1371/journal.pone.0222290 (PMC6746377; doi:10.1371/journal.pone.0222290)
Supplement: S4 Table — (DOCX) [file pone.0222290.s004.docx]

S4 Table. Comparison between Sedia and Maxim between-site and within-site variances of OD measurements for EPs 1-4 (mixed effects model estimates).

|  | | **Model-Based Variance Mean (95% CI)** | |  |
| --- | --- | --- | --- | --- |
| **Sample** | **Variance Type** | **Sedia Kit** | **Maxim Kit** | **Result** |
| LA_0001 | Between Site | 0.0037 (0.0018, 0.0109) | 0.0010 (0.0005, 0.0034) | Variances Equal |
| LA_0002 | Between Site | 0.0312 (0.0160, 0.0858) | 0.0082 (0.0037, 0.0300) | Variances Equal |
| LA_0003 | Between Site | 6.3535 (3.2644, 17.344) | 4.6942 (2.2179, 15.701) | Variances Equal |
| LA_0004 | Between Site | 0.2664 (0.1367, 0.7286) | 0.0456 (0.0210, 0.1619) | Variances Equal |
| LA_0005 | Between Site | 0.6913 (0.3553, 1.8866) | 0.2422 (0.1144, 0.8114) | Variances Equal |
| LA_0006 | Between Site | 3.9947 (1.9976, 11.611) | 2.3980 (1.1316, 8.0459) | Variances Equal |
| LA_0007 | Between Site | 1.4514 (0.7461, 3.9575) | 0.4017 (0.1898, 1.3435) | Variances Equal |
| LA_0008 | Between Site | 1.0972 (0.5639, 2.9934) | 0.2345 (0.1108, 0.7842) | Variances Equal |
| LA_0009 | Between Site | 0.4737 (0.2370, 1.3744) | 0.4233 (0.2000, 1.4161) | Variances Equal |
| **LA_0001** | **Within Site** | **0.0015 (0.0012, 0.0019)** | **0.0001 (0.0001, 0.0002)** | **Sedia Var Higher** |
| **LA_0002** | **Within Site** | **0.0023 (0.0018, 0.0030)** | **0.0052 (0.0040, 0.0071)** | **Maxim Var Higher** |
| LA_0003 | Within Site | 0.0736 (0.0556, 0.1021) | 0.0812 (0.0592, 0.1184) | Variances Equal |
| **LA_0004** | **Within Site** | **0.0145 (0.0120, 0.0178)** | **0.0318 (0.0256, 0.0407)** | **Maxim Var Higher** |
| LA_0005 | Within Site | 0.0019 (0.0011, 0.0037) | 0.0024 (0.0014, 0.0052) | Variances Equal |
| **LA_0006** | **Within Site** | **0.1245 (0.0875, 0.1914)** | **0.0612 (0.0456, 0.0865)** | **Sedia Var Higher** |
| LA_0007 | Within Site | 0.0014 (0.0008, 0.0029) | 0.0028 (0.0016, 0.0061) | Variances Equal |
| LA_0008 | Within Site | 0.0020 (0.0012, 0.0041) | 0.0016 (0.0009, 0.0035) | Variances Equal |
| **LA_0009** | **Within Site** | **0.0335 (0.0272, 0.0423)** | **0.0132 (0.0104, 0.0171)** | **Sedia Var Higher** |
