# Supplementary material for: Development of an international external quality assurance program for HIV-1 incidence using the Limiting Antigen Avidity assay
Source: PLoS One. 2019 Sep 16;14(9):e0222290. doi: 10.1371/journal.pone.0222290 (PMC6746377; doi:10.1371/journal.pone.0222290)
Supplement: S5 Table — (DOCX) [file pone.0222290.s005.docx]

S5 Table. Comparison between Sedia and Maxim between-site and within-site variances of ODn measurements for EPs 1-4 (mixed effects model estimates).

|  | | **Model-Based Variance Mean (95% CI)** | |  |
| --- | --- | --- | --- | --- |
| **ID** | **Variance Type** | **Sedia Kit** | **Maxim Kit** | **Result** |
| LA_0001 | Between Site | 0.6914 (0.3432, 2.0452) | 0.7613 (0.3571, 2.5933) | Variances Equal |
| LA_0002 | Between Site | 0.2391 (0.1213, 0.6716) | 0.3607 (0.1692, 1.2284) | Variances Equal |
| LA_0003 | Between Site | 10.658 (5.4639, 29.240) | 6.0913 (2.8775, 20.382) | Variances Equal |
| **LA_0004** | **Between Site** | **0.0055 (0.0024, 0.0242)** | **0.1815 (0.0837, 0.6467)** | **Maxim Var Higher** |
| **LA_0005** | **Between Site** | **0.3904 (0.1987, 1.0892)** | **0.0187 (0.0084, 0.0730)** | **Sedia Var Higher** |
| LA_0006 | Between Site | 3.5782 (1.7835, 10.481) | 1.2835 (0.6015, 4.3849) | Variances Equal |
| **LA_0007** | **Between Site** | **1.9146 (0.9834, 5.2311)** | **0.0733 (0.0334, 0.2703)** | **Sedia Var Higher** |
| **LA_0008** | **Between Site** | **1.1786 (0.6041, 3.2354)** | **0.0441 (0.0205, 0.1539)** | **Sedia Var Higher** |
| LA_0009 | Between Site | 0.0306 (0.0141, 0.1088) | 0.0129 (0.0055, 0.0566) | Variances Equal |
| **LA_0001** | **Within Site** | **0.0048 (0.0039, 0.0060)** | **0.0005 (0.0004, 0.0006)** | **Sedia Var Higher** |
| **LA_0002** | **Within Site** | **0.0019 (0.0015, 0.0024)** | **0.0085 (0.0065, 0.0116)** | **Maxim Var Higher** |
| **LA_0003** | **Within Site** | **0.4177 (0.3154, 0.5796)** | **0.0813 (0.0592, 0.1184)** | **Sedia Var Higher** |
| **LA_0004** | **Within Site** | **0.0318 (0.0264, 0.0390)** | **0.0573 (0.0460, 0.0732)** | **Maxim Var Higher** |
| LA_0005 | Within Site | 0.0044 (0.0026, 0.0088) | 0.0052 (0.0030, 0.0113) | Variances Equal |
| **LA_0006** | **Within Site** | **0.1528 (0.1073, 0.2350)** | **0.0557 (0.0415, 0.0787)** | **Sedia Var Higher** |
| LA_0007 | Within Site | 0.0029 (0.0017, 0.0061) | 0.0070 (0.0040, 0.0152) | Variances Equal |
| LA_0008 | Within Site | 0.0048 (0.0029, 0.0097) | 0.0032 (0.0018, 0.0071) | Variances Equal |
| LA_0009 | Within Site | 0.0441 (0.0358, 0.0557) | 0.0292 (0.0231, 0.0381) | Variances Equal |
