# Supplementary figures and images for: Development of an international external quality assurance program for HIV-1 incidence using the Limiting Antigen Avidity assay
Source: PLoS One. 2019 Sep 16;14(9):e0222290. doi: 10.1371/journal.pone.0222290 (PMC6746377; doi:10.1371/journal.pone.0222290)

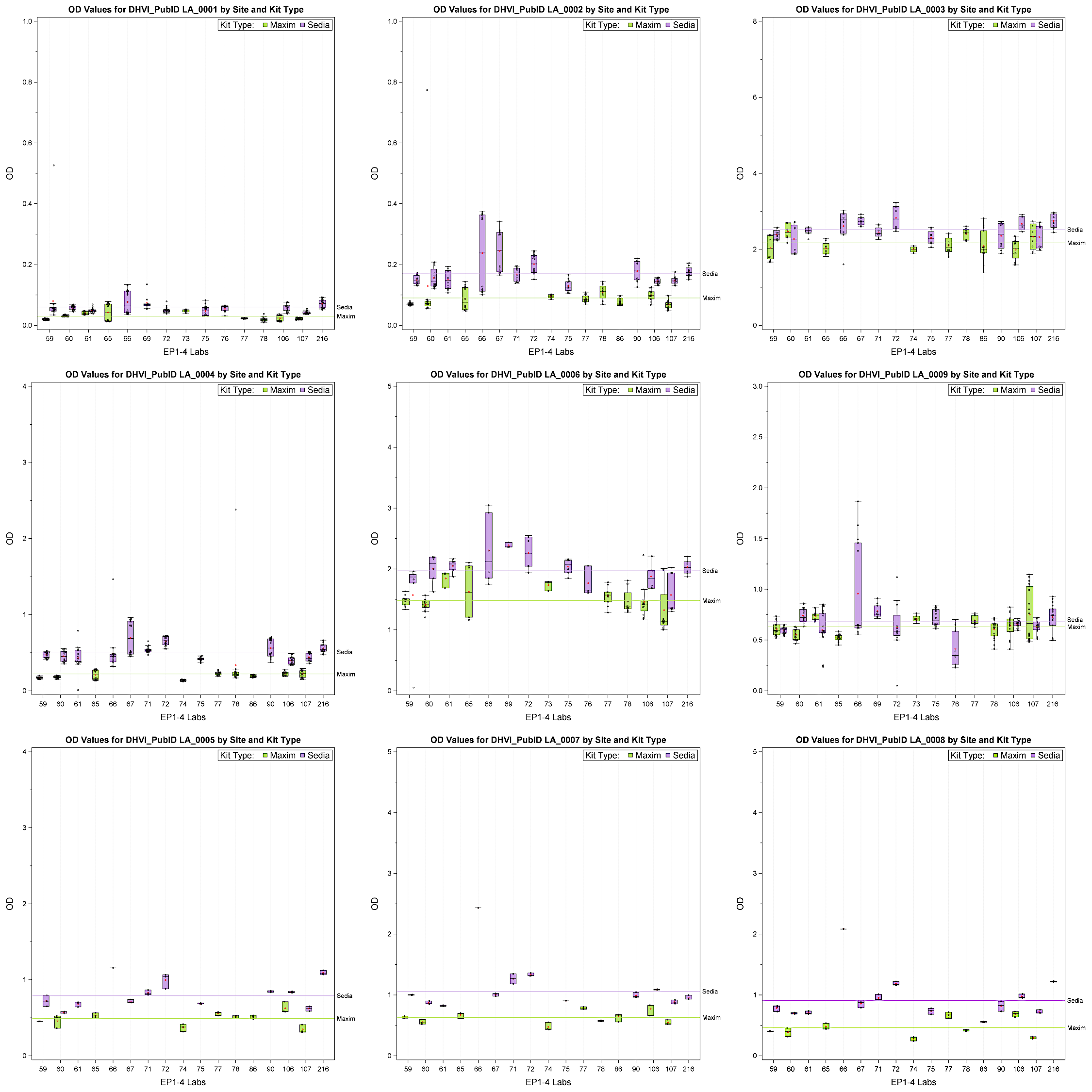

Supplement: S1 Fig — Maxim kits are shown in green and Sedia kits are in purple. Eight out of nine samples had significantly higher Sedia means than Maxim means. (TIF) [file pone.0222290.s006.tif]
